# Intensity-Modulated Radiation Therapy with Simultaneous Integrated Boost for Clinically Node-Positive Prostate Cancer: A Single-Institutional Retrospective Study

**DOI:** 10.3390/cancers13153868

**Published:** 2021-07-31

**Authors:** Masahiro Onishi, Hidemasa Kawamura, Kazutoshi Murata, Tatsuro Inoue, Hiroto Murata, Yosuke Takakusagi, Noriyuki Okonogi, Yu Ohkubo, Masahiko Okamoto, Takuya Kaminuma, Tetsuo Sekihara, Takashi Nakano, Tatsuya Ohno

**Affiliations:** 1 Oncology Center, Hidaka Hospital, Takasaki 370-0001, Japan; 024masahiro@gmail.com (M.O.); tatsuroinoue6789@gmail.com (T.I.); 2 Department of Radiation Oncology, Gunma University Graduate School of Medicine, Maebashi 371-8511, Japan; okamott@gunma-u.ac.jp (M.O.); tohno@gunma-u.ac.jp (T.O.); 3 QST Hospital, National Institutes for Quantum and Radiological Science and Technology, Chiba 263-8555, Japan; murata.kazutoshi@qst.go.jp (K.M.); okonogi.noriyuki@qst.go.jp (N.O.); 4 Department of Radiation Oncology, Saitama Cancer Center, Kita-adachi 362-0806, Japan; murata.hiroto@saitama-pho.jp; 5 Department of Radiation Oncology, Kanagawa Cancer Center, Yokohama 241-8515, Japan; y-takakusagi@kcch.jp; 6 Department of Radiation Oncology, Saku Central Hospital Advanced Care Center, Saku 385-0051, Japan; ohkubo.yu@sakuhp.or.jp; 7 Division of Radiation Oncology, Shibukawa Medical Center, Shibukawa 377-0280, Japan; kaminuma.takuya.kn@mail.hosp.go.jp; 8 Department of Urology, Hidaka Hospital, Takasaki 370-0001, Japan; sekihara_t@hidaka-kai.com; 9 National Institute for Quantum and Radiological Science and Technology, Chiba 263-8555, Japan; nakano.takashi2@qst.go.jp

**Keywords:** prostate cancer, node-positive, IMRT, simultaneous integrated boost, tomotherapy

## Abstract

**Simple Summary:**

Recently, it has been shown that radiation therapy (RT) together with androgen-depletion therapy (ADT) might be more beneficial compared with ADT alone for clinically node-positive (cN1) prostate cancer. However, there are a limited number of studies that have addressed specific RT techniques and analyzed their clinical results. The present study was a retrospective analysis of cN1 prostate cancer patients treated with intensity-modulated radiation therapy with simultaneous integrated boost (SIB-IMRT), in addition to ADT, in our hospital. The present study suggests that ADT plus SIB-IMRT for cN1 prostate cancer treatment was safe and effective, was well tolerated, and had acceptable rates of late toxicity. Further prospective multicenter studies would be required to confirm the robustness of the present results.

**Abstract:**

This study aimed to evaluate clinical outcomes and the toxicity of intensity-modulated radiation therapy with simultaneous integrated boost (SIB-IMRT) combined with androgen-deprivation therapy for clinically node-positive (cN1) prostate cancer. We retrospectively analyzed 97 patients with cN1 prostate cancer who received SIB-IMRT between June 2008 and October 2017 at our hospital. The prescribed dosages delivered to the prostate and seminal vesicle, elective node area, and residual lymph nodes were 69, 54, and 60 Gy in 30 fractions, respectively. Kaplan–Meier analysis was used to determine 5-year biochemical relapse-free survival (bRFS), relapse-free survival (RFS), overall survival (OS), and prostate cancer-specific survival (PCSS). Toxicity was evaluated using the Common Terminology Criteria for Adverse Events ver. 4.0. Over a median follow-up duration of 60 months, the 5-year bRFS, RFS, OS, and PCSS were 85.1%, 88.1%, 92.7% and 95.0%, respectively. Acute Grade 2 genito-urinary (GU) and gastro-intestinal (GI) toxicities were observed in 10.2% and 2.1%, respectively, with no grade ≥3 toxicities being detected. The cumulative incidence rates of 5-year Grade ≥2 late GU and GI toxicities were 4.7% and 7.4%, respectively, with no Grade 4 toxicities being detected. SIB-IMRT for cN1 prostate cancer demonstrated favorable 5-year outcomes with low incidences of toxicity.

## 1. Introduction

The management of lymph node-positive (cN1) prostate cancer remains controversial. Indeed, the presence of lymph node involvement in prostate cancer has been widely considered a poor prognostic factor, with cN1 prostate cancer having been classified as stage IV disease [1], similar to prostate cancer with distant metastases. Therefore, cN1 prostate cancer has been historically managed using noncurative treatment alone, such as androgen-depletion therapy (ADT) [2,3]. In the National Cancer Data Base (NCDB) analysis, the 5-year overall survival (OS) for ADT monotherapy was an unsatisfactory 49% [4]. However, retrospective series and database analyses have recently shown that local therapies, such as radiotherapy (RT) and radical total prostatectomy, together with ADT may be more beneficial for cN1 prostate cancer compared to ADT alone [4,5,6,7,8,9,10,11,12,13], while certain guidelines have recommended ADT plus RT for cN1 prostate cancer [14,15,16]. However, given that previous studies have provided insufficient information regarding specific RT techniques for cN1 prostate cancer, the optimal target and dose fractionation has remained unclear. Although evidence has shown that intensity-modulated radiation therapy (IMRT) can be effective for localized or locally advanced prostate cancer [17,18], only a few studies regarding IMRT for cN1 prostate cancer are available. Considering that our hospital has been performing IMRT with simultaneous integrated boost (SIB-IMRT) in addition to ADT for cN1 prostate cancer, the current study sought to retrospectively analyze the efficacy and safety of ADT plus SIB-IMRT for patients with cN1 prostate cancer admitted at our hospital.

## 2. Materials and Methods

### 2.1. Patient Selection

A total of 97 consecutive patients with cN1 prostate cancer who received definitive RT at our hospital between June 2008 and October 2017 were retrospectively analyzed. All patients were histologically confirmed to have adenocarcinoma of the prostate. Pelvic lymph node involvement was clinically diagnosed using computed tomography (CT) and magnetic resonance imaging (MRI) based on the size and shape, as well as response to ADT. All patients were classified using the TNM Classification according to the International Union against Cancer TNM Classification of Malignant Tumors, 7th edition. Patients with distant metastases, including nonregional lymph node metastases, were excluded. Written informed consent was obtained from all patients prior to treatment.

### 2.2. Target Delineation

Axial CT images of the pelvis with a 3 mm slice thickness were acquired using a 16-row multi-detector CT scanner (Aquilion LB, Toshiba Medical, Otawara, Japan). T2-weighted MR images of the pelvic with a 3 mm slice thickness were obtained and merged with the planning CT images to delineate the target volumes. Thereafter, the target volumes and organs at risk (OAR) were contoured on the FocalSim version 4.3.1 (Focal, Eindhoven, Netherlands) or Pinnacle 3 (Philips Radiation Oncology Systems, Fitchburg, MA, USA) treatment planning systems. The clinical target volume of the prostate (CTV_prostate) comprised the entire prostate and the proximal seminal vesicles in general. However, for locally advanced cases, such as T3a, T3b, and T4, the CTV_prostate was expanded to include the entire prostate, seminal vesicles, and the invasive portion. The planning target volume of the prostate (PTV_prostate) was defined as the CTV_prostate plus a 5 mm margin in all directions except posteriorly, where a 3 mm margin was used. The GTV of the lymph nodes (GTV_LN) was defined as enlarged lymph nodes upon initial diagnosis that remained identifiable on the CT simulation scans after neoadjuvant ADT. The CTV of the lymph nodes (CTV_LN) was equivalent to the GTV_LN. The PTV lymph node (PTV_LN) was defined as the CTV_LN plus a 5 mm margin in all directions. Regional lymph nodes included obturator, internal iliac, external iliac, and presacral (down to S3) lymph nodes. In cases where lymph nodes were located near the internal and external iliac bifurcation, the elective lymph node area was expanded by adding a 7 mm margin to the internal iliac, external iliac, and obturator vessels. The subclinical PTV (PTV_sub) was generally defined as the elective lymph node area with a 2 mm margin. The bladder, rectum, sigmoid, and femoral heads were displayed as a solid structure defined by the outer wall. The rectum was extended from the anal canal to the rectosigmoid flexure. The intestine was delineated as the whole intestinal cavity encompassing the small bowel and colon.

### 2.3. Intensity-Modulated Radiation Therapy Planning

The CT datasets and structures were transferred to the TomoTherapy Treatment Planning system (Accuray Inc. Sunnyvale, CA, USA) for inverse planning. The prescribed dosages delivered to the PTV_prostate, PTV_LN, and PTV_sub were 69 Gy (2.3 Gy per fraction), 60 Gy (2.0 Gy per fraction), and 54 Gy (1.8 Gy per fraction) in 30 fractions over 6 weeks using the SIB technique, respectively. For the area of overlap between the PTV_LN and the bowel, however, the prescribed dosage was reduced to 54 Gy. The dosages given to the PTV_prostate (69 Gy) and PTV_sub (54 Gy) in 30 fractions were biologically equivalent to 74.91 and 50.91 Gy, respectively, assuming an α/β of 1.5 Gy for prostate cancer. The objective of each plan was to deliver the prescribed dosage to 95% of the PTV (D95%), limiting the maximum dose to <105% of the prescribed dosage to the PTV. The dose–volume constrains for the OAR were as follows: rectum V60 Gy (percentage of the rectum volume receiving at least 60 Gy) <17%, V37 < 35%, and V21 < 60%; bladder V60.5 < 25% and V37.8 < 50%. The prescription indicated that the dose be reduced as low as possible for the intestine. The following planning parameters were used to generate the plans: field width of 2.48 cm, modulation factor of 2.0, and pitch of 0.287. A fine grid (2.7 × 2.7 mm) was used for the final calculation process after satisfying all constrains. Two or more radiation oncologists examined all contoured structures and treatment planning to ensure consistency in radiation treatment planning.

### 2.4. Positioning and Treatment

Patients were asked to maintain a comfortably filled bladder and a completely empty rectum to reduce discrepancies in bladder and rectal volumes between simulation and treatment. All patients underwent simulation and treatment in the supine position. A heel support was used for immobilization, while laser marks on the patients’ skin were used to reduce errors in pitch and yaw rotation. All patients received helical tomotherapy under daily image-guided radiation therapy (IGRT). Before each fraction, mega voltage CT images were superimposed onto the treatment plans to verify the positional layout and preparation of the patients. Automatic registration was applied based on bony anatomy, followed by manual adjustment in the lateral, antero-posterior, and cranio-caudal directions to match the prostate and lymph nodes.

### 2.5. Androgen Deprived Therapy

ADT primarily consisted of combined androgen blockade (CAB) therapy using a luteinizing hormone-releasing hormone (LH-RH) analog and an oral anti-androgen. Almost all patients underwent neoadjuvant ADT for more than 6 months prior to radiation therapy. Adjuvant ADT after radiation therapy was prescribed at the discretion of the urologist, with the treatment duration remaining undefined.

### 2.6. Follow-Up

Patient follow-up was scheduled 1 month after the end of RT, every 3 months for the first 3 years, and then every 3–12 months thereafter. PSA levels were measured before RT and during each follow-up examination. CT images were collected at least once per year after RT to assess for any relapse or metastatic progression. Patients with biochemical failure underwent imaging studies, such as CT, MRI, and bone scan. Genito-urinary (GU) and gastro-intestinal (GI) toxicities were classified using the Common Terminology Criteria for Adverse Events ver. 4.0 (CTCAEv4.0, NCI Common Terminology Criteria for Adverse Events (CTCAE) V4.0 Data Files. Available online: http://evs.nci.nih.gov/ftp1/CTCAE/About.html accessed on 26 September 2020). Acute toxicities were defined as symptoms occurring during treatment and up to 3 months after RT. Late toxicities were defined as symptoms occurring >3 months after RT. Toxicity events were defined as symptoms higher in grade compared to their respective baseline levels. The maximal recorded grade for each symptom was defined as the toxicity grade. Rectal and intestinal toxicities presenting during or after RT were evaluated separately. Patients with suspected rectal bleeding underwent endoscopic examination, during which rectal toxicities were confirmed.

### 2.7. Statistical Analyses

Biochemical relapse-free survival (bRFS), relapse-free survival (RFS), prostate cancer-specific survival (PCSS), and OS curves were calculated using Kaplan–Meier analysis. Survival endpoints were measured from the first day of radiation therapy. Biochemical failure was defined according to the Astro/Phoenix criteria (PSA nadir + 2 ng/mL). RFS included locoregional relapse or distant failure detected on imaging studies. Patients who survived without recurrence were censored during the last follow-up. To calculate PCSS, death due to prostate cancer was defined as that due to castration-resistant distant metastases or PSA relapse, with no other obvious cause of death. Univariate analysis using the log-rank test was conducted to identify prognostic factors for OS and bRFS. Cumulative incidences of GU and GI were analyzed. All statistical analyses were performed using SPSS software (version 24, IBM corp., Armonk, NY, USA), with *p* < 0.05 indicating statistical significance.

## 3. Results

A total of 97 consecutive patients with cN1 prostate cancer who underwent definitive RT between June 2008 and October 2017 were analyzed. Details regarding patient and treatment characteristics are summarized in Table 1 and Table 2. Accordingly, patients had a median age of 69 years (range, 48–83 years), received a median follow-up of 60 months (range, 5–125 months), and had a median initial PSA level of 33.0 ng/mL (range 3.6–948.1 ng/mL). Most of the patients had locally advanced disease (87.6% with T3-4) and a Gleason Score of 8 to 10 (85.6%). According to the Roach formula based on the Gleason Score and PSA, the estimated risk of lymph node involvement was high, with a median of 51.3%. All patients received both neoadjuvant and adjuvant ADT for a median of 6 months (range, 3–22 months) and 54 months (range, 2–126 months), respectively. Most of the patients (94.8%) received CAB therapy with an LH-RH analog and anti-androgen. The median PSA level prior to RT was 0.14 ng/mL (range, 0–14.52 ng/mL; data for five patients were unavailable). Two patients who had elevated PSA during neoadjuvant ADT were deemed castration-resistant. Meanwhile, the 71 patients (73.2%) who had radiologically residual lymph node involvement received nodal boost to 60 Gy using SIB. The median CTV_LN was 0.79 cm^3^ (range, 0.09–12.67 cm^3^).

**Figure 1:**
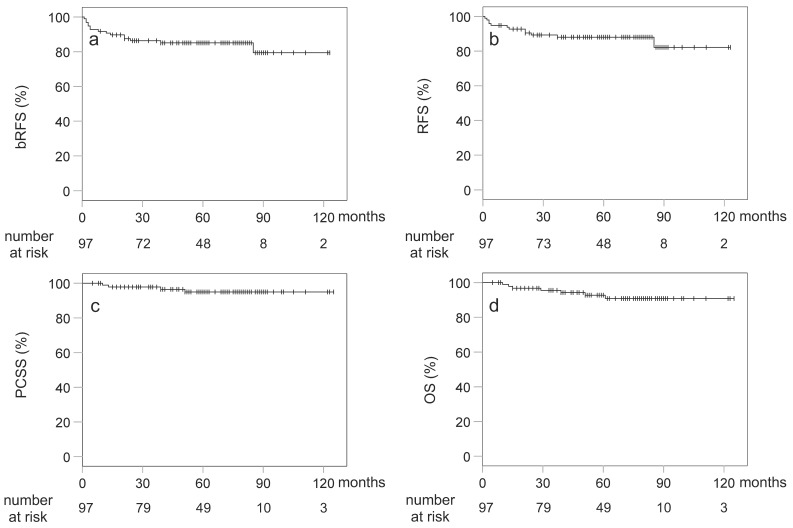
Kaplan–Meier curves for (**a**) biochemical relapse-free survival (bRFS); (**b**) relapse-free survival (RFS); (**c**) overall survival (OS); (**d**) prostate cancer-specific survival (PCSS).

The included patients had a 5-year bRFS, RFS, OS, and PCSS of 85.1, 88.1%, 92.7%, and 95.0%, respectively (Figure 1). Univariate analysis found no factors significantly correlated with 5-year bRFS and OS (Table 3). During the follow-up period, 15 patients (15.5%) experienced biochemical failure, with the duration from RT to biochemical failure ranging from 1 to 85 months (median of 13 months). Moreover, 12 patients (12.4%) developed clinical recurrence, including 2 (2.1%) with locoregional disease, 8 (8.2%) with distant disease, and 2 (2.1%) with both locoregional and distant disease, at the time of recurrence. All four of the locoregional recurrences were local, while one was a lymph node with nodal boost. Among the 10 patients with out-of-field recurrences, 7 involved the bone, 4 involved distant lymph nodes, 2 involved the lungs, and 2 involved the liver. Although three patients experienced biochemical failure, their localization could not be determined. Overall, seven patients (7.2%) died during the follow-up period, among which four (4.1%) were due to prostate cancer and two (2.1%) to comorbidities (lung cancer or chronic renal failure), with one (1.0%) unknown cause of death.

The maximal GU and GI toxicities are detailed in Table 4. No Grade ≥ 3 acute GU or GI toxicities, or toxicity-related treatment interruptions, occurred. However, 10 patients (10.2%) experienced acute Grade 2 GU toxicities, whereas 2 patients (2.1%) experienced acute Grade 2 GI toxicities. The majority of the acute GI toxicities comprised Grade 1 diarrhea in 23 patients (23.7%), and Grade 2 diarrhea in 2 patients (2.1%).

**Figure 2:**
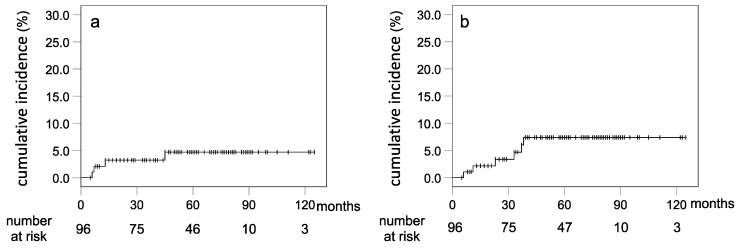
(**a**) Cumulative incidence of Grade ≥ 2 genito-urinary toxicity; (**b**) cumulative incidence of Grade ≥ 2 gastrointestinal toxicity.

Late toxicities were assessed in 96 patients, excluding 1 for whom no data were available due to death within 3 months. Accordingly, four patients (4.2%) exhibited late Grade 2 GU toxicity, such as dysuria, among whom only one had hematuria. The 5-year actuarial risk for developing late Grade ≥2 GU toxicity was 4.7% (Figure 2). Moreover, five patients (5.2%) exhibited late Grade 2 GI toxicities, while one (1.0%) suffered from Grade 3 toxicity. Most of the late GI toxicities involved rectal hemorrhage, while acute diarrhea was mostly common during remission. The 5-year actuarial risk for developing late Grade ≥ 2 GI toxicity was 7.4% (Figure 2).

## 4. Discussion

The present study describes the long-term outcomes of SIB-IMRT for cN1 prostate cancer. The present study showed that SIB-IMRT combined with ADT had favorable outcomes in patients with cN1 prostate cancer, a finding consistent with those of previous studies (Table 5). Some single-institutional retrospective studies have also been conducted [5,6,7]. In a retrospective single-institution study, Zagars et al. [5] showed that patients with biopsy-proven node-positive (pN1) prostate cancer, who received RT combined with ADT, had better 5-year OS compared with those treated with ADT alone (92% vs. 82%). All patients in the said study had subclinical, node-positive, after-staging lymphadenectomy, which is a different cohort from the present study of clinically diagnosed, node-positive prostate cancer, but with comparable survival rates. Although no randomized control trials have been conducted, several population-based studies have been published on RT for node-positive patients. Node-positive prostate cancer treated with RT showed better OS and PCSS compared with node-positive prostate cancer that received no local treatment, based on two retrospective analyses of the surveillance, epidemiology, and end results (SEER) database [8,9]. Tward et al. [8] reported that patients who received RT (either brachytherapy or external beam RT) had better 5-year OS (67% vs. 56%; *p* < 0.01) and PCSS (78% vs. 71%; *p* < 0.01) vs. patients who received no RT. Another SEER analysis by Rusthoven et al. [9] showed that patients who received RT had better 10-year OS (45% vs. 29%; *p* < 0.01) and PCSS (67% vs. 53%; *p* < 0.01) vs. patients who received no local therapy. Of note, the SEER data do not have information on ADT and do not definitively distinguish between patients with clinical and pathological node-positive cancer. The NCDB analysis by Lin et al. [4] showed that cN1 prostate cancer patients treated using RT combined with ADT had better 5-year OS compared with patients treated using ADT alone (72% vs. 49%; *p* < 0.01). James et al. [10] published an analysis of cN1 patients from the control arm of the Systemic Therapy in Advancing or Metastatic Prostate Cancer: Evaluation of Drug Efficacy (STAMPEDE) trial. This exploratory analysis showed that patients who received RT combined with ADT had better 2-year failure-free survival (81% vs. 53%). These retrospective and database analyses suggest that RT combined with ADT for N1 prostate cancer improves survival and cancer control compared with ADT alone. The outcomes of the present study were consistent with the results of these population-based studies. The disadvantage of these database analyses is the insufficient information regarding specific RT techniques, RT fields, and dose fractionation. The advantages of the present study are that a relatively homogeneous RT technique was used, and specific RT fields and dose fractions were presented.

Several RCTs have shown the benefits of local dosage escalation for localized prostate cancer [19,20,21,22,23,24]. Furthermore, a single-institution series has shown that local dosage escalation to 72 Gy or higher can improve bRFS among patients with cN1 prostate cancer [25]. In line with the aforementioned findings, the present study employed local dosage escalation to 74.9 GyEQD2, with our results showing local recurrence in only five cases (5.2%) and a favorable 5-year bRFS of 85.1%. Notably, the patients included herein received boost irradiation of 60 Gy delivered to the residual lymph nodes after neoadjuvant ADT. Guidelines for cN1 prostate cancer recommend a dosage escalation, such as 60 GyEQD2 or higher, for lymph node involvement, while maintaining dosage constraints in the surrounding normal organs [14,15]. Considering that only one patient (1.0%) developed lymph node recurrence which was boosted, the dosages utilized in the present study can be considered reasonable.

The efficacy of pelvic irradiation to the elective lymph node area for cN1 prostate cancer [26,27], as well as its optimal target and dosage, remain unclear. Based on the consensus guidelines for contouring the elective lymph node area in prostate cancer, published by the Radiation Therapy Oncology Group (RTOG) and PIVOTAL study group [28,29], the present study included the obturator, internal iliac, external iliac, and presacral lymph node areas. Although the common iliac area was generally excluded, the elective lymph node area was expanded in cases with lymph node involvement near the internal and external iliac bifurcation. Meanwhile, recent studies reporting on the distribution and recurrence patterns of lymph node involvement in prostate cancer have shown significant lymph node involvement outside the target volume indicated by the aforementioned conventional guidelines, with particularly high occurrences in the common iliac and para-aortic regions [30,31,32]. Therefore, it is possible that the target volume utilized in the present study may not have covered some of the involved lymph nodes. However, among the 10 patients included herein who had recurrences outside the target volume, none had common iliac lymph node involvement, 4 had para-aortic lymph node involvements, and 3 had distant metastases, including bone metastases. This result suggests that only certain patients would benefit from the inclusion of the common iliac and para-aortic area in the target volume. Considering the potential for increasing toxicity due to larger target volumes, routinely including the common iliac and para-aortic regions for cN1 prostate cancer may not be necessary.

The elective pelvic nodal irradiation dosage utilized herein was 50.9 GyEQD2, which was equivalent to that employed in RCTs on definitive RT for prostate cancer (45–50.4 GyEQD2) [33,34,35,36,37,38,39,40]. However, a dosage of approximately 45–50 GyEQD2 has been considered insufficient for controlling potential prostate cancer lesions. On the other hand, the fact that only 1 patient (1.0%) included in the present study developed recurrence from within the target volume, whereas 10 (10.3%) developed recurrences outside the target volume, suggests that the dosage also reduced the risk of the recurrence. Nonetheless, further follow-up is needed to determine whether the pelvic target and dosage used herein were appropriate.

Nevertheless, concerns have been raised regarding the possibility of increased toxicity with local dose escalation or elective pelvic nodal irradiation. In fact, the RTOG study 94–13 showed Grade 2 acute GU and GI toxicity rates of 31% and 47% among patients who received pelvic irradiation with 3D-CRT, respectively [37,41]. IMRT allows for a highly uniform dose distribution with a steep dose gradient between the target and normal organs. Moreover, its combination with daily IGRT can reduce the treatment margins associated with internal motion and layout uncertainty, allowing the delivery of high dosage concentrations to the target while reducing toxicity delivered to the other organs [42,43,44,45,46]. Indeed, Engel et al. reported Grade 2 and 3 acute GI toxicity rates of 7% and 0%, and Grade 2 and 3 acute GU toxicity rates of 14% and 4%, respectively, following whole pelvic RT with SIB-IMRT and IGRT for high-risk and node-positive prostate cancer [47]. Moreover, a phase I study had shown that dose-escalated IMRT including elective pelvic lymph nodes was safe, and had acceptable late GU and GI toxicity [48,49]. The present study had favorable toxicity results that were comparable to those reported in previous studies on pelvic irradiation with IMRT for prostate cancer.

All patients included herein received ADT combined with RT, with the median duration of neoadjuvant and adjuvant ADT being 6 and 54 months, respectively. Indeed, a number of studies have recommended ADT in combination with RT for cN1 prostate cancer [14,15]. Moreover, a subset analysis of patients with node-positive prostate cancer in the RTOG study 85–31, a randomized trial on RT alone versus RT plus ADT for patients with clinical T3 or node-positive prostate cancer, found that combination therapy promoted a substantial improvement in PFS and OS [50]. Nonetheless, opinions have been divided regarding the optimal systemic therapy to combine with RT for cN1 prostate cancer, and particularly whether to incorporate abiraterone into ADT [51]. Although the 2020 NCCN guideline suggests either ADT alone or ADT plus abiraterone [14], the optimal duration of ADT combined with RT for cN1 prostate cancer was not clearly established, while their recommendations were based on estimates from studies on high-risk prostate cancer [52]. Moreover, while the 2020 NCCN guideline recommends long-term ADT over at least 2–3 years [14], the 2019 Australian and New Zealand Radiation Oncology Genito-Urinary group recommends a duration of 18–36 months based on comorbidity, ADT tolerance, and tumor burden [15]. Notably, the median duration of ADT in the present study was longer than that indicated in the guidelines, which may have allowed us to control potential lesions both inside and outside the target volume. Despite the favorable outcomes of the current study, further studies are needed to confirm the effects of long-term ADT combined with RT for cN1 prostate cancer on complications and survival.

The current study has several limitations worth noting. First, the diagnosis of lymph node involvement may be inadequate. Pelvic lymph node dissection is considered the most reliable procedure for accurate nodal staging [53]. However, contrast-enhanced CT and conventional MRI using size and morphology as diagnostic criteria have a sensitivity of 40% and a specificity of about 80% for lymph node involvement, respectively, which are not sufficiently reliable [54]. In the present study, although the diagnosis of lymph node involvement was based on the clinical evaluation of the response to neoadjuvant ADT in addition to conventional imaging, this method may be inadequate, considering the lack of pathological evaluation. Recent biological imaging modalities, such as prostate-specific membrane antigen positron emission tomography (PSMA)/CT, have been reported as non-invasive nodal staging methods, with a pooled analysis sensitivity for the initial staging of lymph node detection of 77%, and a specificity of 97% [55]. Such a new imaging technique would be able to properly evaluate the efficiency of SIB-IMRT. Second, given that this was a retrospective, single-institution study, unknown biases could have affected our results. Furthermore, the current study group was too small to be conclusive as regards the superiority of SIB-IMRT over conventional or other RT techniques. As such, a multicenter prospective study would be needed to confirm the robustness of our results.

## 5. Conclusions

The present retrospective analysis suggests that ADT plus SIB-IMRT for cN1 prostate cancer was safe and effective, was well tolerated, and had acceptable rates of late toxicity.

## Figures and Tables

**Table 1:** Patient characteristics.

Characteristics (*n* = 97)	Value
Age at treatment (years), median (range)	69 (48–83)
ECOG performance status (%)	
0	71 (73.2)
1	26 (26.8)
≥2	0
Initial PSA level (ng/mL), median (range)	33.0 (3.6–948.1)
Tumor stage (%)	
T1-2	12 (12.4)
T3a	30 (30.9)
T3b	39 (40.2)
T4	16 (16.5)
Gleason Score (%)	
6	3 (3.1)
7	11 (11.3)
8	23 (23.7)
9	54 (55.7)
10	6 (6.2)
Predicted risk of pelvic lymph node involvement (Roach formula), median (range)	51.3 (2.7–100)
PSA level prior to RT (ng/mL), median (range)	0.14 (0–14.52)
CRPC (%)	
Yes	2 (2.1)
No	95 (97.9)
Diabetes (%)	
Yes	15 (15.5)
No	82 (84.5)
Anticoagulants (%)	
Yes	11 (11.3)
No	86 (88.7)

Abbreviations: ECOG, eastern cooperative oncology group; PSA, prostate specific antigen; CRPC, castration-resistant prostate cancer.

**Table 2:** Treatment characteristics.

Characteristics (*n* = 97)	Value
Follow-up period (months), median (range)	60 (5–125)
Androgen-deprived therapy (%)	97 (100.0)
Combined androgen blockade	92 (94.8)
LH-RH analogue	4 (4.1)
Anti-androgen	1 (1.0)
Duration period (months), median (range)	66 (17–133)
Neoadjuvant ADT (%)	
Yes	97 (100.0)
No	0
Duration period (months), median (range)	6 (3–22)
Adjuvant ADT	
Yes	97 (100.0)
No	0
Duration period (months), medina (range)	54 (2–126)
Lymph nodal boost (%)	
Yes	71 (73.2)
No	26 (26.8)
Volume of CTV_LN (cc), median (range)	0.79 (0.09–12.67)

Abbreviations: ADT, androgen-deprived therapy.

**Table 3:** Univariate analysis for factors associated with bRFS and OS.

Characteristics (*n* = 97)	*n*	5 y bRFS (%)	*p* Value	5 y OS (%)	*p* Value
T stage					
1, 2, 3a	42	90.3	0.352	97.2	0.367
3b, 4	55	81.1		89.1	
Gleason Score					
8<	14	100	0.097	100	0.249
≥8	83	82.5		91.4	
iPSA					
20<	34	79.3	0.084	95.7	0.792
≥20	63	90.2		91.4	
Lymph nodal boost					
No	26	91.3	0.212	96	0.987
Yes	71	82.7		91.6	

Abbreviations: bRFS, biochemical relapse-free survival; OS, overall survival.

**Table 4:** The incidence levels of toxicities according to the common terminology criteria for adverse events, version 4.0.

Toxicity	G0	G1	G2	G3
Acute toxicity (*n* = 97) (%)				
GU	24 (24.7)	63 (64.9)	10 (10.3)	0
GI	63 (64.9)	32 (33.0)	2 (2.1)	0
Diarrhea	71 (73.2)	23 (23.7)	2 (2.1)	0
Late toxicity (*n* = 96) (%)				
GU	57 (59.4)	35 (36.5)	4 (4.2)	0
Hematuria	92 (95.8)	3 (3.1)	1 (1.0)	0
GI	73 (76.0)	17 (17.7)	5 (5.2)	1 (1.0)
Rectal hemorrhage	75 (78.1)	15 (15.6)	5 (5.2)	1 (1.0)
Diarrhea	95 (99.0)	1 (1.0)	0	0

Abbreviations: GU, genitourinary; GI, gastrointestinal.

**Table 5:** Previous studies on prostate cancer treatments.

Study	Study Design	No. of Patients	Median Follow-Up (*y*)	Primary Treatment	Treatment Detail	ADT	Outcome	No RT	RT
Present study	Retrospective, single-institution	97	5.0	ADT + RT	EBRT, prostate + pelvis	All patients	5 y OS 5 y PCSS 5 y bRFS 5 y RFS		93% 95% 85% 88%
Zagars et al., 2001 [5]	Retrospective, single-institution	pN1: 255	ADT: 9.4 ADT+RT: 6.2	ADT vs. ADT + RT	EBRT, prostate	All patients	5 y OS 10 y OS	83% 46%	92% 67% (*p* = 0.008)
Fonteyne et al., 2009 [6]	Retrospective, single-institution	cN1: 25 pN1:55	3.0	ADT + RT	EBRT, prostate + pelvis	All patients	3 y bRFS 3 y RFS		81% 89%
Mallick et al., 2019 [7]	Retrospective, single-institution	61	4.0	ADT + RT	EBRT, prostate + pelvis	All patients	4 y OS 4 y RFS		91% 77.5%
Tward et al., 2013 [8]	Retrospective, population based, SEER	1100	7.5	No RT vs. RT	EBRT, brachytherapy	N/A	5 y OS 5 y PCSS	56% 71%	68% (*p* < 0.01) 78% (*p* < 0.01)
Rusthoven et al., 2014 [9]	Retrospective, population based, SEER	796	6.8	No RT vs. RT	EBRT	N/A	10 y OS 10 y PCSS	29% 53%	45% (*p* < 0.01) 67% (*p* < 0.01)
Lin et al., 2015 [4]	Retrospective, population based, NCDB	636	2.7	ADT vs. ADT + RT	EBRT	All patients	5 y OS	49%	72% (*p* < 0.01)
James et al., 2015 [10]	Prospective, multi-institutions, exploratory analysis	157	N/A	ADT vs. ADT + RT	EBRT, prostate ± pelvis	All patients	2 y FFS	53%	81%

Abbreviations: ADT, androgen-deprived therapy; RT, radiation therapy; EBRT, external beam radiotherapy; OS, overall survival; PCSS, prostate cancer-specific survival; bRFS, biochemical relapse-free survival; RFS, relapse-free survival; FFS, failure-free survival.

## Data Availability

The data presented in this study are available on request from the corresponding author. The data are not publicly available due to restrictions of patients’ privacy or ethical aspect.
